# Stress–Pollution Interactions: An Emerging Issue in Children’s Health Research

**DOI:** 10.1289/ehp.119-a430

**Published:** 2011-10-01

**Authors:** Catherine M. Cooney

**Affiliations:** Catherine M. Cooney, a science writer based in Washington, DC, has written for *Environmental Science & Technology* and *Chemical Watch*.


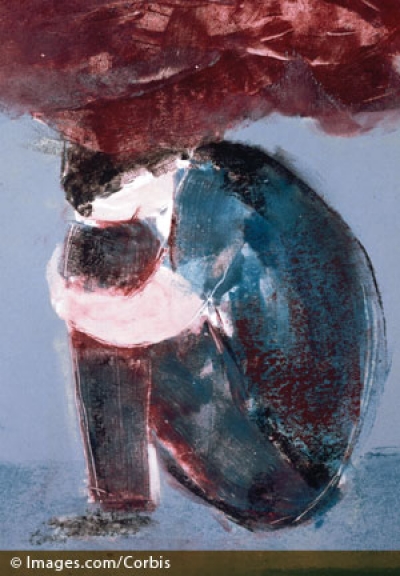
Cumulative risk assessment posits that multiple agents work together to induce disease and that multiple stressors therefore must be considered in order to gain a true understanding of why adverse health effects occur.^1^ Now a small but growing number of scientists are pushing the envelope by investigating whether chronic psychological stress might be one of those factors, enhancing a child’s vulnerability to certain chemical exposures and contributing to effects that later show up as asthma, neurodevelopmental disorders, cardiovascular disease, obesity, cancer, and other problems. These researchers are also starting to identify biomarkers that may shed light on the mechanisms by which psychological stress acts on a child’s developing immune system and brain to modify or enhance the response to certain pollution exposures such as traffic-related air pollutants and lead.

“We really don’t know how broadly such interactions may occur across chemicals. They are much more likely to occur when the chemical itself acts directly upon stress systems,” says Deborah Cory-Slechta, a professor of environmental medicine at the University of Rochester School of Medicine & Dentistry.

“We know some chemicals that interact with stress, such as lead exposure, but we don’t know which others do.”

Observations of links between stress and disease date back to at least the twelfth century, when the philosopher Maimonides cited emotional upset as a factor in asthma.^2^ But proving such links poses a significant challenge, says Malcolm P. Cutchin, a professor at the School of Medicine of the University of North Carolina at Chapel Hill. “Much has been hypothesized about the linkages, but we are just now beginning to tease out relationships and understand the processes,” Cutchin says. As researchers have learned more about techniques that can identify chemical and stress exposures in the human body, they have begun to apply techniques to estimate how people respond to stress and how that response, if it goes awry, can facilitate the development of diseases.

## What Is Stress?

Some stress is good. Human systems are designed with an autonomic nervous system that responds to stress by stimulating the release of the hormones adrenaline (to speed the heat rate, pump up blood pressure, and mobilize energy) and cortisol (to replenish energy supplies and prime the immune system to combat bacterial, viral, or injury threats). This added energy urges children to keep trying to crawl until they can reach a shiny toy sitting across the floor and allows teens to think through a complicated geometry quiz question. This type of stress has been called “positive stress.”^3^

“Tolerable stress” falls under the category of more intense short-term experiences, such as the departure of a loved one or a natural disaster. The body’s response systems rev up to respond to the event but are still able to shut down once the experience ends. Stress is termed “toxic” when it is prolonged, severe, and/or frequent. For children this may involve ongoing physical or emotional abuse, chronic neglect, caregiver substance abuse or mental illness, or exposure to violence, experienced without adequate adult support.^3^

Yet there are people who experience chronic stress as children and grow into resilient adults, indicating that the degree of a person’s stress response depends on many things, including genetic factors, personality characteristics, and learned coping skills. “Studies^4^^,^^5^ do show that cumulative stress can cause you to be more adaptable,” Cory-Slechta says. For reasons not fully understood, she says, “Some people tend to do better in stressful situations as adults because they have experienced a higher level of stress earlier in childhood.”

**Figure:**
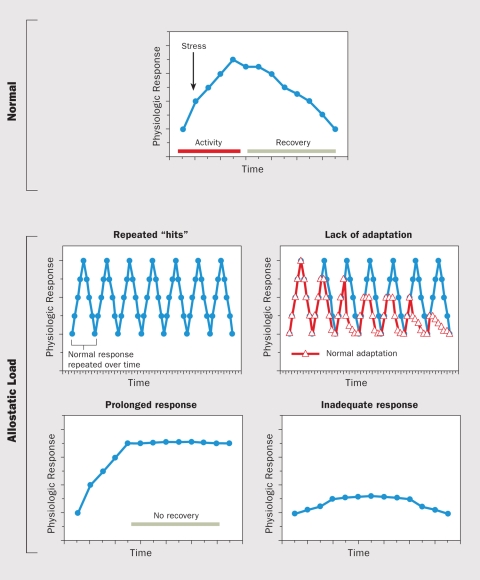
A normal allostatic response (top panel), once initiated by a stressor, is sustained for an appropriate period and then turned off. Four abnormal conditions (bottom panels) can lead to allostatic load: 1) repeated “hits” from multiple stressors, 2) a lack of adaptation (“wearing down” over time), 3) a prolonged stress response, and 4) an inadequate response, which can lead to compensatory hyperactivity of other body systems. Source: McEwen (1998)^34^

Generally speaking, however, if the “fight or flight” response brought on by stress continues for too long, a constant flow of the hormones may “reset” the immune system so that it either stays revved up or becomes suppressed—that is, it is no longer in the optimal state of balance that promotes good health.^6^ For example, the ability of the immune system to be stabilized by cortisol may no longer work, and the production of cytokines and mobilization of inflammatory cells may increase,^6^ potentially contributing to problems such as obesity, cancer, and coronary heart disease.

“The mechanisms are not totally clear; in other words, we are not exactly sure how stress works on the body to create future health problems. But we are getting close,” says Richard Hunter, a research associate at Rockefeller University’s Harold and Margaret Milliken Hatch Laboratory of Neuroendocrinology. There is growing evidence that stress may influence one or more of the same physiological pathways as certain chemical toxicants, potentially including oxidative stress, proinflammatory immune function, and autonomic disruption.^7^

There is unlikely to be only one predominant pathway for stress effects on the body or on pollution susceptibility, says Jane E. Clougherty, an assistant professor in the University of Pittsburgh Department of Environmental and Occupational Health. “Stress is a nonspecific constellation of physiological effects, some of which may increase and others decrease individual responsivity to pollution, dependent on the pollution in question and its dose, the chronicity and intensity of stress, target organs and health outcomes of interest, resources, and other individual and community-level factors.”

Clougherty explains there are differences between acute and chronic stress. Acute stress, lasting hours or days, involves release of the hormone cortisol and activation of the hypothalamic–pituitary–adrenal (HPA) axis, whereas chronic stress, lasting weeks or years, involves altered glucocorticoid responsivity and immune, endocrine, and metabolic function.^8^ “It is primarily chronic stress that is hypothesized to increase individuals’ susceptibility to pollution,” Clougherty says. “Aspects of acute stress such as bronchodilation can actually temporarily mask pollution response.”

**Figure:**
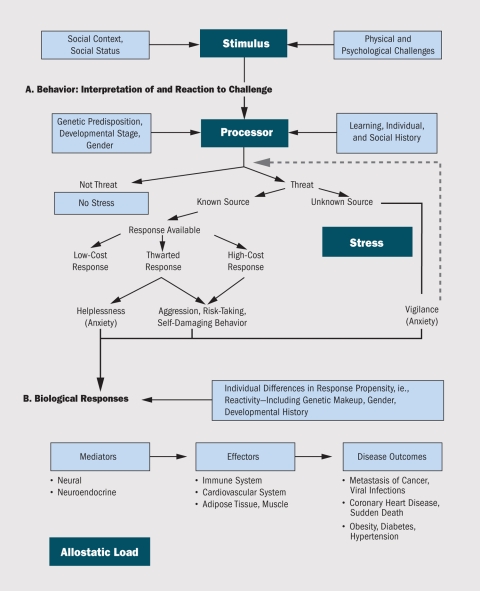
The individual is influenced by genetic makeup, stage of biological development, sex, past learning, and social history in 1) interpreting a physical or psychological stimulus (e.g., as a threat or nonthreat, as a known or unknown threat, as a threat for which a coping mechanism is or is not available) and 2) responding to the stimulus. If the source of a potential threat is unknown, the individual stays in a state of aroused vigilance until the decision can be made that a threat does or does not exist (dotted arrow). If the source of a potential threat is known, then chooses a response, if one is available. “Low-cost” responses are not stressful or damaging to the individual whereas “high-cost” responses (e.g., thrill-seeking, risk-taking, or self-abusive behaviors) can take a physical or psychological toll on the individual. If the available response is thwarted, the individual may feel helpless or frustrated, or engage in displaced aggression or other high-cost responses. The autonomic nervous system, hypothalamic–pituitary–adrenal (HPA) axis, and cardiovascular, endocrine, and immune systems launch responses to perceived threats. Allostatic load—the wear and tear induced when these responses are sustained over time—is being linked to a number of disease outcomes. Source: McEwen and Stellar (1993)^10^

That’s not the limit of the complexities of the interaction. “The relative temporality of stress and pollution exposures matter, as well as intensity,” Clougherty says; that is, generally stress must have occurred before a chemical exposure in order for an adverse interaction to be observed. Moreover, stress–pollution interactions may not necessarily be linear. Clougherty says, “Sometimes one exposure matters, sometimes the other, sometimes both. And sometimes either exposure is high enough that the system is ‘saturated,’ and interactions no longer matter.”^9^

In 1993 Bruce S. McEwen and Eliot Stellar proposed a biological framework concept called “allostatic load” as a way to visualize factors that influence how an individual interprets and responds to stress.^10^ “Allostasis” refers to the adaptive state of responding to a challenge; allostatic load is the cumulative physical impact of that adaptation. As McEwen later described it, allostatic load is “the price the body pays for being forced to adapt to adverse psychosocial or physical situations, and it represents either the presence of too much allostasis or the inefficient operation of the allostasis response systems, which must be turned on and then turned off again after the stressful situation is over.”^11^ However, it remains difficult to use the concept of allostatic load across disciplines, because there is no uniform agreement on the relative importance of the various stressors that should be factored in to its calculation.

## Let’s Go to the Lab

In research published in June 2010, Clougherty and colleagues reported that respiratory effects of laboratory rats’ exposures to air pollution were exacerbated by stress.^12^ The team found that bloodborne markers of systemic inflammation were elevated under chronic stress and that only with the combined exposures of chronic stress and high concentrated ambient particulate exposures did the rats exhibit a rapid, shallow breathing pattern. The paper is thought to be the first toxicologic study to illustrate the role of stress in amplifying an animal’s respiratory response to particulate matter air pollution, and it offers a first step toward identifying pathways through which chronic stress may influence the animal’s susceptibility to pollution, Clougherty says.

Many researchers focused on projects that examine the potential link between stress, pollution, and adverse health outcomes in humans have studied impoverished families living in inner cities. Cory-Slechta, who examines the health effects of lead exposures combined with stress, says one hypothesis that has drawn researchers to this field of study suggests that children living in low-income communities in urban settings are even more vulnerable to certain illnesses because of the high levels of environmental exposures they face (e.g., peeling lead-based paint, diesel exhaust, and emissions from nearby industrial facilities). Individuals in these communities also tend to have higher incidences of disease, Cory-Slechta says. “One hypothesis out there is that these people are also living in higher-stress communities, and they have higher levels of stress hormones,” she says.

This may particularly be true among urban populations with relatively frequent exposure to neighborhood crime and violence.^13^^,^^14^ This was initially explored in an article published by Wright and colleagues detailing four case studies of urban pediatric patients with asthma, which illustrated an association between a child’s exposure to violence and exacerbation of asthma symptoms.^15^

“Sometimes there wasn’t an environmental trigger for the asthma exacerbations like we are used to seeing, such as high-ozone days,” Wright says of the patient histories in the paper. “It really did seem to be an emotional stressor that was exacerbating the asthma, in this case being violence in the community. You see that clinically—that there are emotional triggers to children’s asthma. Researchers have seen it with other disorders that have an immune basis as well, like inflammatory bowel disease or arthritis.” Since then, she says, a number of published studies have corroborated these anecdotal relationships.^16^^,^^17^^,^^18^ Wright’s group has also demonstrated elevated cortisol levels in school-aged children growing up in more violent neighborhoods.^19^

In 2007 Clougherty, Wright, and colleague Jonathan Levy of Harvard School of Public Health published a study that examined children’s exposure to traffic-related air pollution and to urban violence (as measured by the frequency with which parents reported their children witnessing incidences of physical assault, shootings, stabbings, or domestic verbal abuse, or hearing gunshots). Children exposed to high levels of urban violence experienced stronger health effects of traffic-related air pollution, as indicated by asthma onset.^20^ In separate work, Edith Chen, codirector of the Psychobiology of Health Laboratory at the University of British Columbia, and colleagues similarly showed an association between high levels of self-reported family stress and greater effects of traffic-related pollution on adverse asthma outcomes in adolescents, including greater production of asthma-related inflammatory markers.^21^

Other pioneering work examining the interaction of stress and air pollution drew data from the University of Southern California (USC) Children’s Health Study, a longitudinal study of respiratory health among children in 13 Southern California communities.^22^ Robert McConnell, deputy director of the USC Children’s Environmental Health Center, and colleagues followed 2,497 children with no history of respiratory problems over three years. They collected information on exposure to traffic-related pollution and whether the children had been exposed to tobacco smoke *in utero*. By the end of three years they found the risk of developing asthma associated with traffic-related pollution was significantly higher for children of parents reporting high levels of personal stress. Stress, as well as low parental education, also was associated with heightened response to prenatal exposure to tobacco smoke.^23^

Yet researchers point out that family stress and the accompanying anxiety and fearfulness that leads to toxic stress in many children are not limited to low-income communities or to any particular racial group. A seminal 1998 study linking childhood household dysfunction and adult health problems surveyed mostly white middle-class adults, McEwen says. Of the 8,506 respondents, just over half (52.1%) were women, 79.4% were white, and 43% had graduated from college.^24^ Participants who were white or Asian or who had graduated from college did tend to have experienced fewer different kinds of adverse childhood experiences (e.g., physical abuse, sexual abuse, imprisonment of a family member), but it would be a mistake to assume these or any other groups are immune to toxic stress. “You can easily image how living in crowding and poverty and so on would increase the frequency of parents taking things out on their kids, but [family strife] is not absent in the middle class,” McEwen says.

“Both epidemiological and animal studies show that stress may impact key regulatory systems in the body, throwing them out of balance,” Wright says.^7^^,^^25^^,^^26^ “This can happen at any period in life, but if it occurs in a critical stage of development when rapid changes are already taking place—like pregnancy or adolescence—it might have particularly measurable as well as lasting effects.”

One body of research suggests stress exposure *in utero* may contribute to prenatal programming of adult disease. In a special communication published in 2009, McEwen and coauthors Jack P. Shonkoff and W. Thomas Boyce proposed a process in which the fetus “‘reads’ key characteristics of its environment and prepares to adapt to an external world that can vary dramatically in its levels of safety, sufficiency, and peril.”^27^ The developing fetus draws from its mother’s experiences, and if stress hormones are frequently released in the mother, they prepare the fetus for a life outside the womb that is likely to involve high stress. The baby’s systems “retain that initial programming and put the stress response system on a short-fuse and high-alert status,” the three wrote. “Under such circumstances, the benefits of short-term survival may come at a significant cost to longer-term health.”^27^

Other investigators have found the same. Research published in 1998 used paired mother–fetus cortisol measurements to demonstrate a linear relationship between fetal and maternal concentrations.^28^ A study from the following year suggested that prolonged elevation of maternal cortisol may negatively affect the growth of the fetal brain.^29^ And a 2005 review examined evidence that exposure to excessive cortisol *in utero* can disrupt early brain development by interfering with the buildup of neurons and with the maturation of synapses in some brain regions.^30^

## Where Next?

This year the U.S. Environmental Protection Agency (EPA) gave a boost to researchers struggling to define interactions between stress and pollution exposures. In January 2011 the agency awarded seven Science to Achieve Results (STAR)^31^ research grants totaling $7 million to work on new approaches to further the understanding of how stress modifies environmental exposures.^32^ In announcing the grants, Paul Anastas, assistant administrator for the EPA Office of Research and Development, emphasized that the grantees are mindful of concerns raised by community and environmental justice advocates, which include an inability of poor urban communities to participate in the permitting process of industrial facilities in their neighborhood, crowding, family strife, and community violence. Environmental justice advocates add that government rules and regulations don’t consider this disproportionate burden and therefore don’t fully protect the health of these residents.

The STAR grants went to researchers working on new analytical techniques and methodologies that should make future study of cumulative risk easier to conduct and the findings more easily compared. The teams will delve into both societal and environmental factors including strategies for assessing cumulative effects of chemical and nonchemical stressors, cumulative risk assessments in urban populations and low-income communities near a Superfund site, the combined effects of metals and stress on central nervous system function, disparities in air pollutant risks, and the effects of stress and traffic pollutants on childhood asthma.

Carolyn Raffensperger, executive director of the nonprofit Science and Environmental Health Network, says her group is convening a national Cumulative Impacts Working Group to advance research and political awareness of cumulative impacts.^33^ The group includes state and national regulatory agencies (including EPA staffers who are managing the STAR grants), nonprofit organizations, and academicians. Raffensperger says the working group will focus on the legal aspects of regulation and policy that don’t currently consider multiple pollution risks. “The problem with research and the law is that we have an approach that looks at exposures chemical by chemical and [industrial] plant by plant,” she says. “We almost never make a policy decision based on all of the exposures in a person’s environment.”

## References

[1] Levy JI (2008). Is epidemiology the key to cumulative risk assessment?. Risk Anal.

[2] Rosner F. (1981). Moses Maimonides’ *Treatise on Asthma*.. Thorax.

[3] Toxic Stress Response: The Facts [website]. Cambridge, MA:Center on the Developing Child, Harvard University (2011). Available: http://tinyurl.com/3nzcqna [accessed 13 Sep 2011].

[4] Meaney MJ, Szyf M (2005). Maternal care as a model for experience-dependent chromatin plasticity?. Trends Neurosci.

[5] Francis DD, Meaney MJ (1999). Maternal care and the development of stress responses.. Curr Opin Neurobiol.

[6] Miller GE (2002). Chronic psychological stress and the regulation of pro-inflammatory cytokines: a glucocorticoid-resistance model.. Health Psychol.

[7] Wright RJ (2009). Moving towards making social toxins mainstream in children’s environmental health.. Curr Opin Pediatr.

[8] Clougherty JE, Kubzansky LD (2009). A framework for examining social stress and susceptibility to air pollution in respiratory health.. Environ Health Perspect.

[9] Clougherty JE (2006). A longitudinal analysis of the efficacy of environmental interventions on asthma-related quality of life and symptoms among children in urban public housing.. J Asthma.

[10] McEwen BS, Stellar E (1993). Stress and the individual. Mechanisms leading to disease.. Arch Intern Med.

[11] McEwen BS (2002). Sex, stress and the hippocampus: allostasis, allostatic load, and the aging process.. Neurobiol Aging.

[12] Clougherty JE (2010). Chronic social stress and susceptibility to concentrated ambient fine particles in rats.. Environ Health Perspect.

[13] Wright RJ (2006). Health effects of socially toxic neighborhoods: the violence and urban asthma paradigm.. Clin Chest Med.

[14] Lederbogen F (2011). City living and urban upbringing affect neural social stress processing in humans.. Nature.

[15] Wright RJ, Steinbach SF (2001). Violence: an unrecognized environmental exposure that may contribute to greater asthma morbidity in high risk inner-city populations.. Environ Health Perspect.

[16] Sternthal MJ, et al. Community violence and urban childhood asthma: a multilevel analysis. Eur Respir J 36(6):1400–1409 (2010); http://dx.doi.org/10.1183/09031936.00003010. 10.1183/09031936.00003010 PMC4811341 20413538

[17] Gupta RS (2010). The association between community crime and childhood asthma prevalence in Chicago.. Ann Allergy Asthma Immunol.

[18] Swahn MH, Bossarte RM (2006). The associations between victimization, feeling unsafe, and asthma episodes among US high-school students.. Am J Public Health.

[19] Suglia SF (2010). Posttraumatic stress symptoms related to community violence and children’s diurnal cortisol response in an urban community-dwelling sample.. Int J Behav Med.

[20] Clougherty JE (2007). Synergistic effects of traffic-related air pollution and exposure to violence on urban asthma etiology.. Environ Health Perspect.

[21] Chen E (2008). Chronic traffic-related air pollution and stress interact to predict biologic and clinical outcomes in asthma.. Environ Health Perspect.

[22] Shankardass K Parental stress increases the effect of traffic-related air pollution on childhood asthma incidence. Proc Natl Acad Sci USA 106 30); 12406 12411 2009 http://dx.doi.org/10.1073/pnas.0812910106 19620729 10.1073/pnas.0812910106 PMC2718368

[23] Wright RJ (2002). Psychologic stress and asthma: Wright’s response.. Environ Health Perspect.

[24] Felitti VJ (1998). Relationship of childhood abuse and household dysfunction to many of the leading causes of death in adults: the Adverse Childhood Experiences (ACE) Study.. Am J Prev Med.

[25] Fowden AL, et al. Intrauterine programming of physiological systems: causes and consequences. Physiol 21(1):29–37 (2006); http://dx.doi.org/10.1152/physiol.00050.2005. 10.1152/physiol.00050.2005 16443820

[26] Welberg LAM, Seckl JR (2001). Prenatal stress, glucocorticoids and the programming of the brain.. J Neuroendocrinol.

[27] Shonkoff JP (2009). Neuroscience, molecular biology, and the childhood roots of health disparities.. JAMA.

[28] Gitau R (1998). Fetal exposure to maternal cortisol.. Lancet.

[29] Sandman CA. Maternal corticotropin-releasing hormone and habituation in the human fetus. Develop Psychobiol 34(3):163–173 (1999); http://dx.doi.org/ 10.1002/(SICI)1098-2302(199904)34:3<163::AID-DEV1>3.0.CO;2-9. 10.1002/(sici)1098-2302(199904)34:3<163::aid-dev1>3.0.co;2-9 10204092

[30] Van den Bergh BR, Mulder EJ, Mennes M, Glover V (2005). Antenatal maternal anxiety and stress and the neurobehavioural development of the fetus and child: links and possible mechanisms: a review.. Neurosci Biobehav Rev.

[31] The EPA’s STAR program supports human health, ecology, economics, and engineering sciences with grants aimed to support cutting-edge research.

[32] EPA. EPA Awards $7 Million to Study Effects of Pollution Exposures and Social Stressors on Communities. Research grants aim to gather comprehensive community wide data on human health impacts [press release]. Washington, DC:U.S. Environmental Protection Agency (11 Jan 2011). Available: http://tinyurl.com/4xjhy55 [accessed 13 Sep 2011].

[33] Cumulative Impacts Project [website]. Science & Environmental Health Network, Collaborative on Health and the Environment (updated 13 Sep 2011). Available: http://tinyurl.com/65v9gkk [accessed 13 Sep 2011].

[34] McEwen BS (1998). Protective and damaging effects of stress mediators.. New Engl J Med.

